# Supplementary figures and images for: A simple mechanism for integration of quorum sensing and cAMP signalling in Vibrio cholerae
Source: eLife. 2023 Jul 6;12:RP86699. doi: 10.7554/eLife.86699 (PMC10328515; doi:10.7554/eLife.86699)

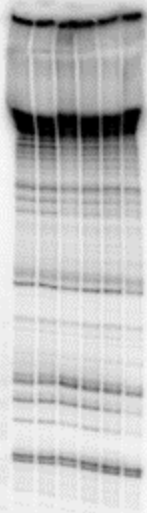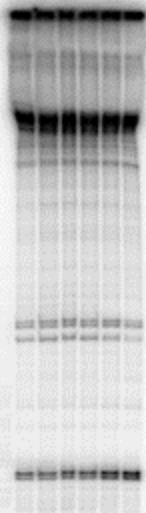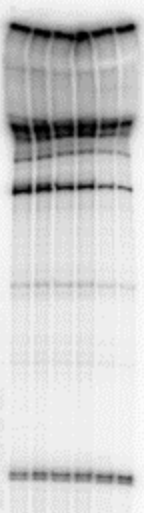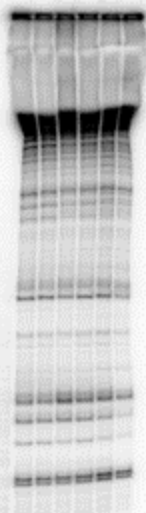

Supplement: Figure 2—source data 1. [file elife-86699-fig2-data1.pdf]

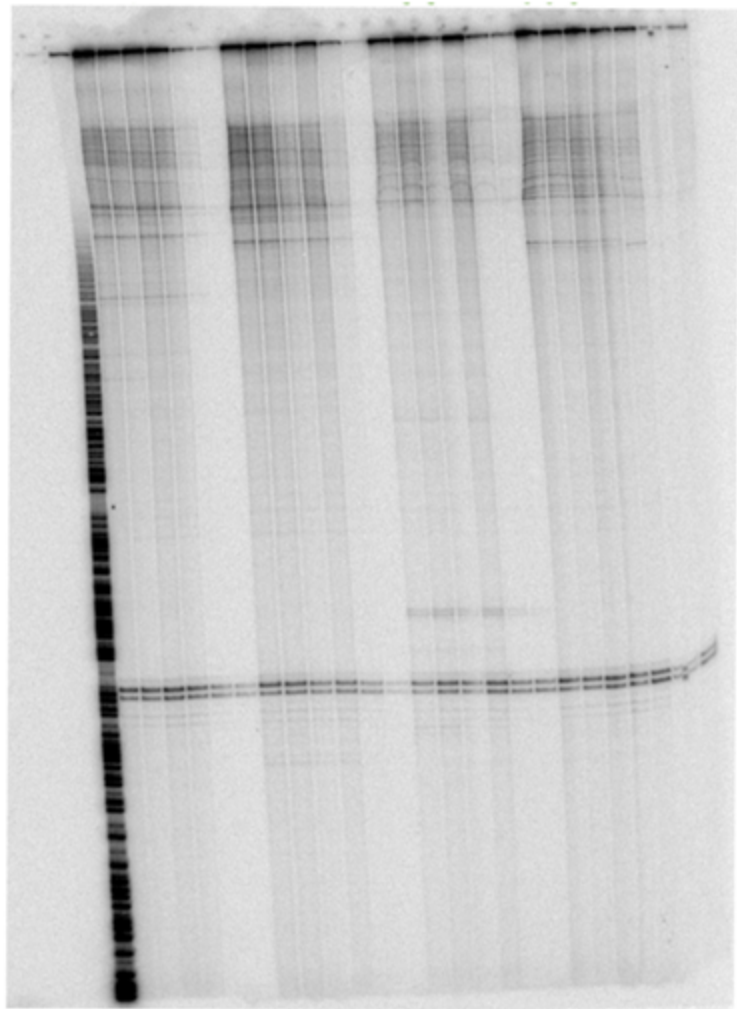

Supplement: Figure 2—source data 2. [file elife-86699-fig2-data2.pdf]

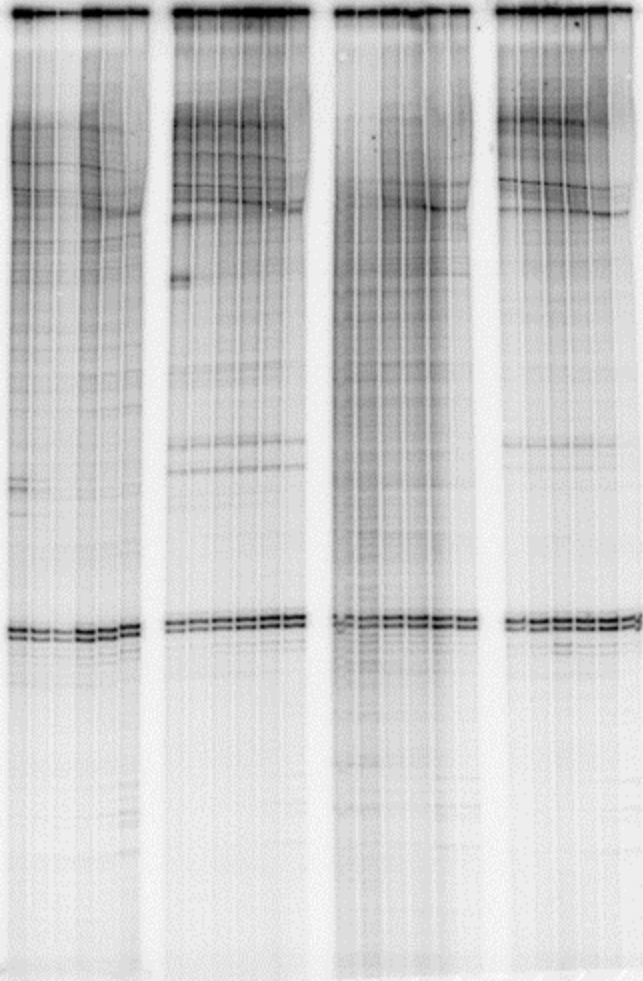

Supplement: Figure 2—source data 3. [file elife-86699-fig2-data3.pdf]

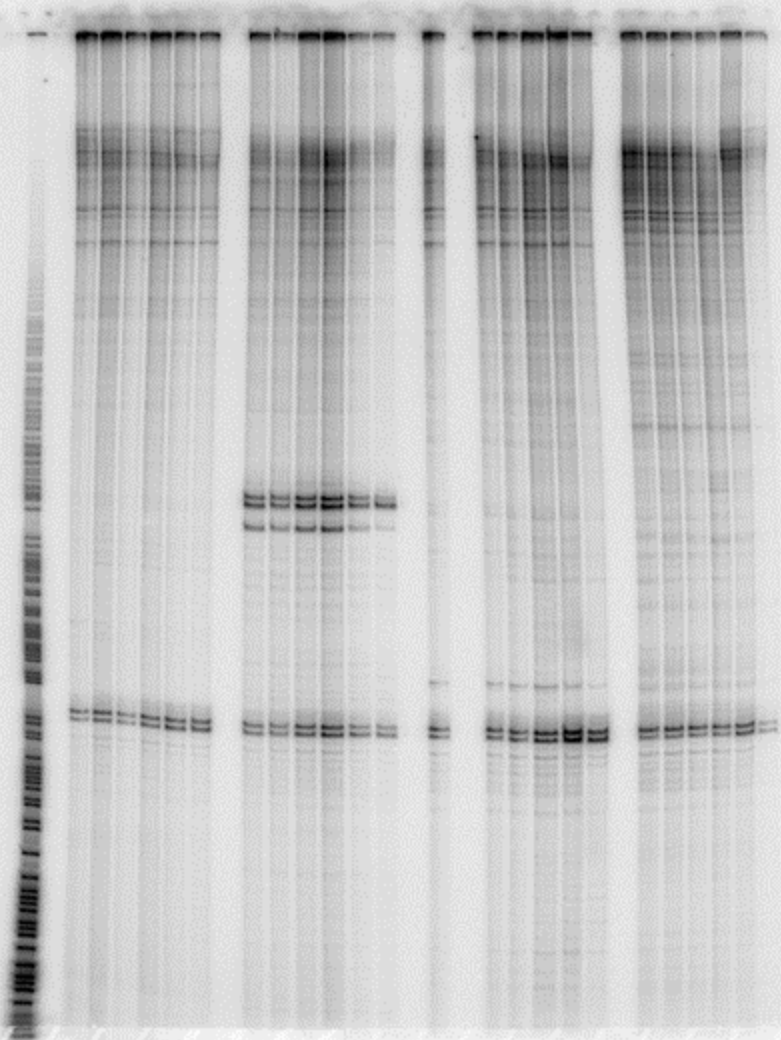

Supplement: Figure 2—source data 4. [file elife-86699-fig2-data4.pdf]

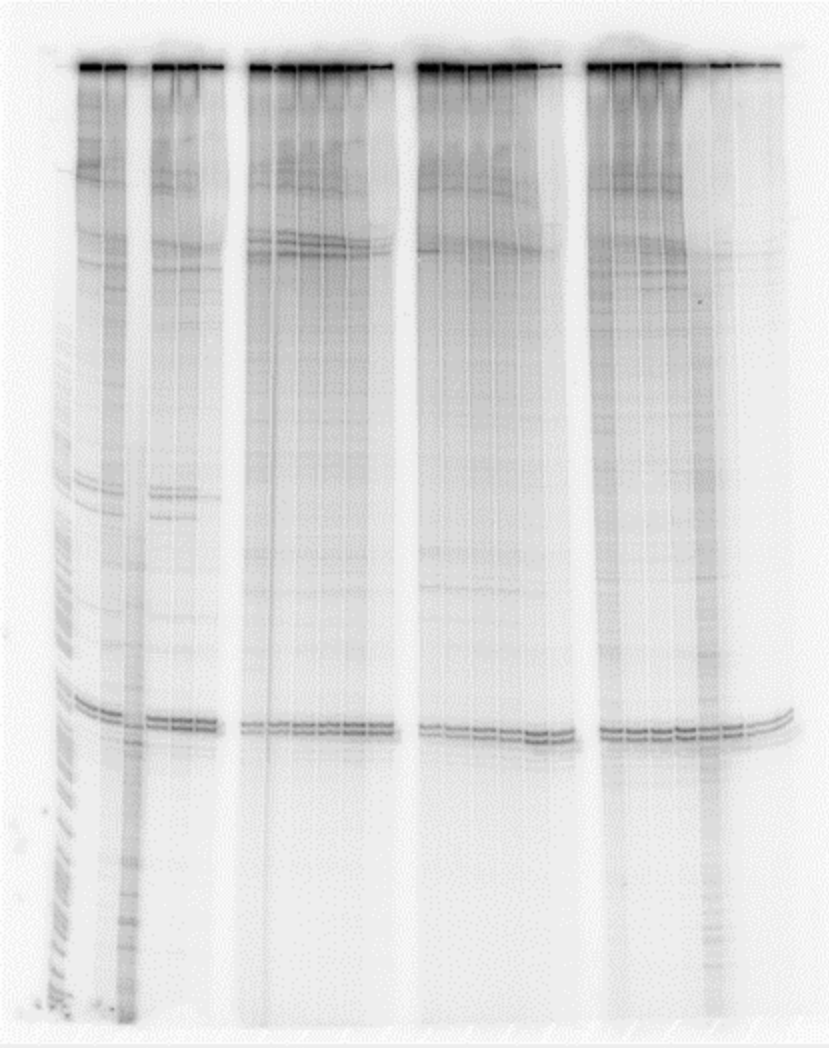

Supplement: Figure 2—source data 5. [file elife-86699-fig2-data5.pdf]

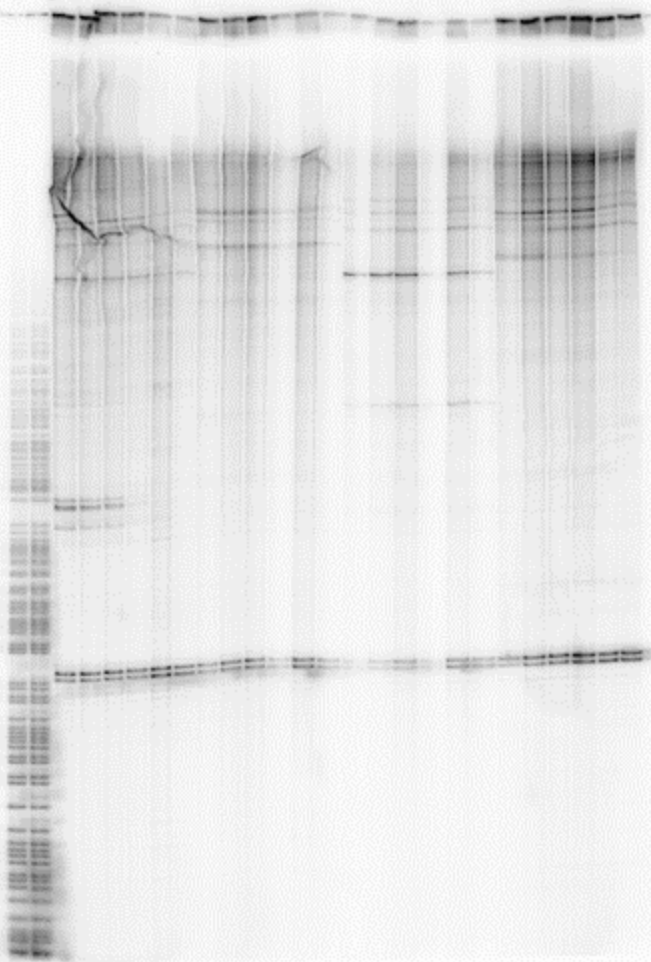

Supplement: Figure 2—source data 6. [file elife-86699-fig2-data6.pdf]

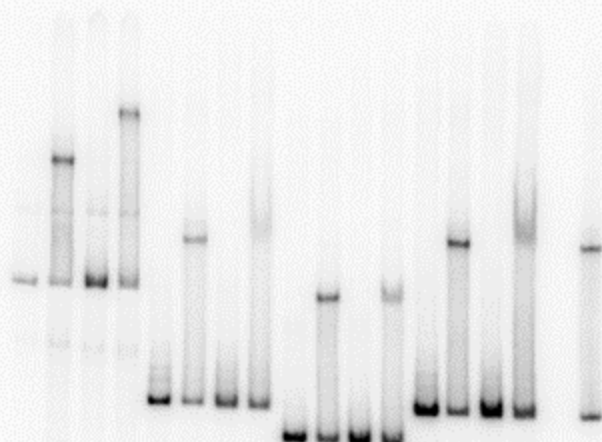

Supplement: Figure 3—source data 1. [file elife-86699-fig3-data1.pdf]

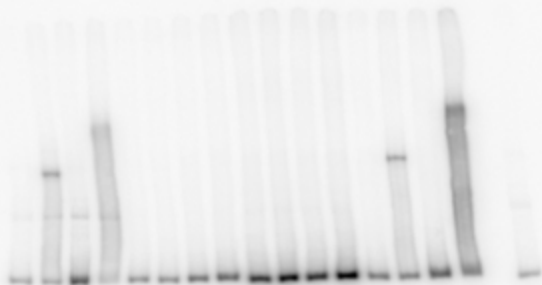

Supplement: Figure 3—source data 2. [file elife-86699-fig3-data2.pdf]

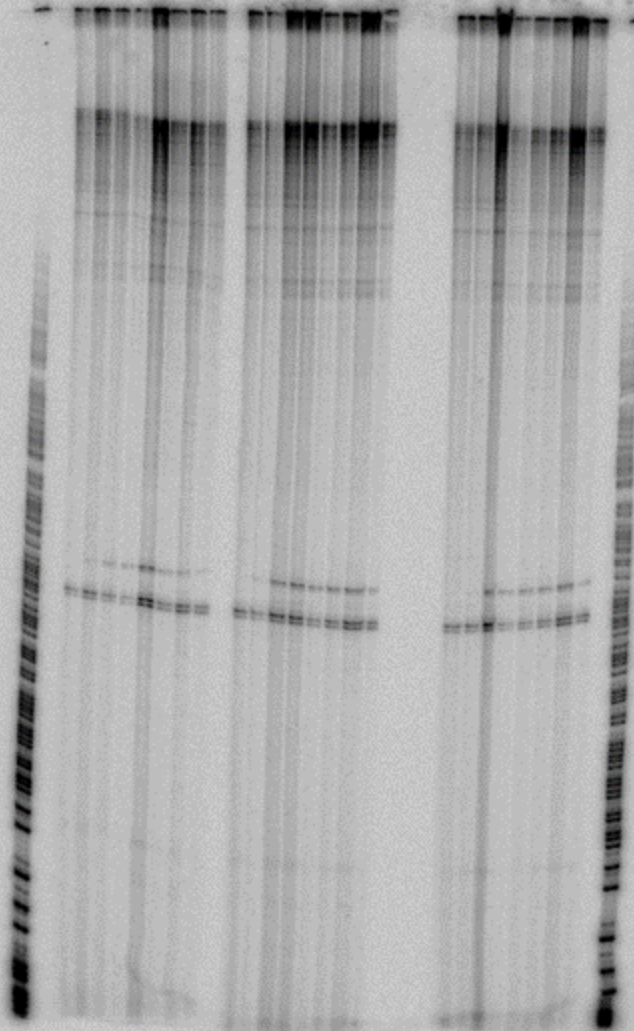

Supplement: Figure 3—source data 3. [file elife-86699-fig3-data3.pdf]

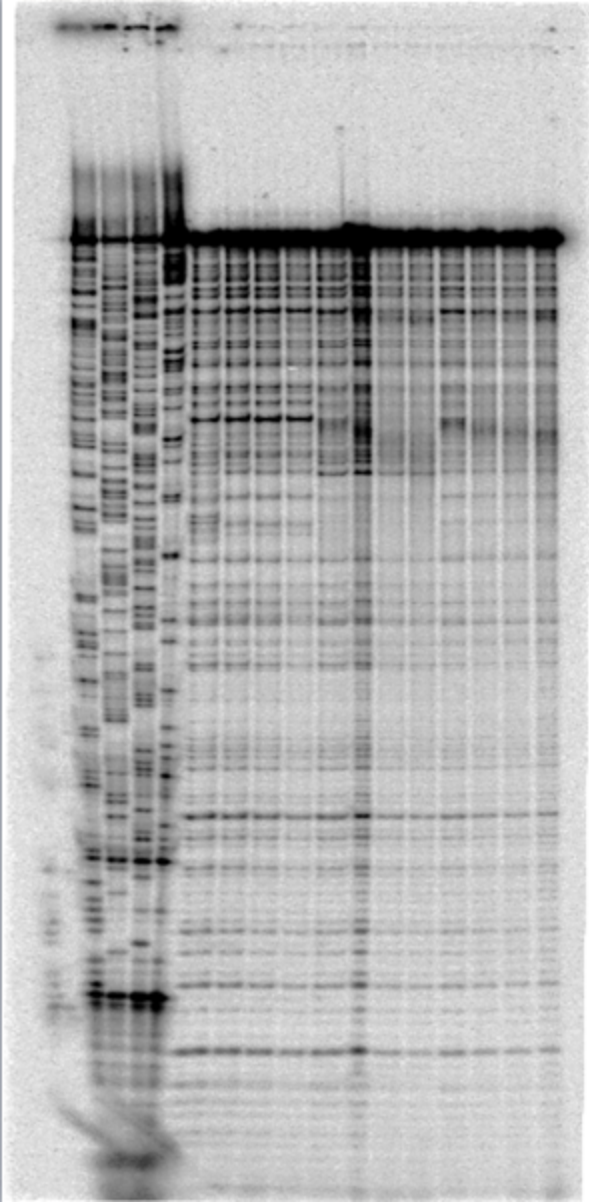

Supplement: Figure 4—source data 1. [file elife-86699-fig4-data1.pdf]

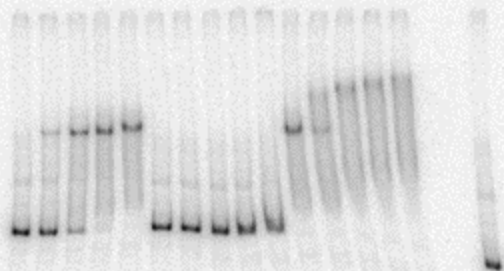

Supplement: Figure 4—source data 2. [file elife-86699-fig4-data2.pdf]

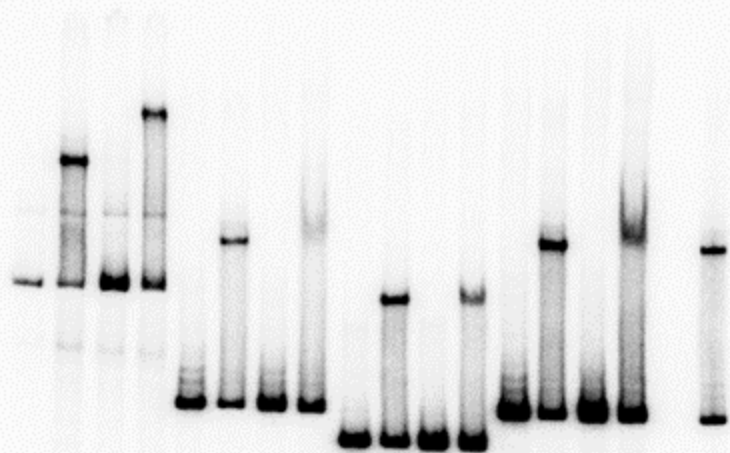

Supplement: Figure 4—source data 3. [file elife-86699-fig4-data3.pdf]

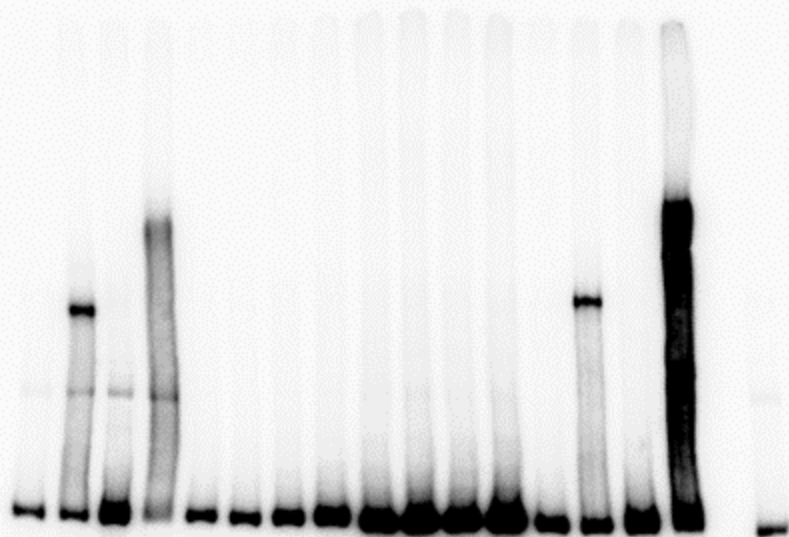

Supplement: Figure 4—source data 4. [file elife-86699-fig4-data4.pdf]

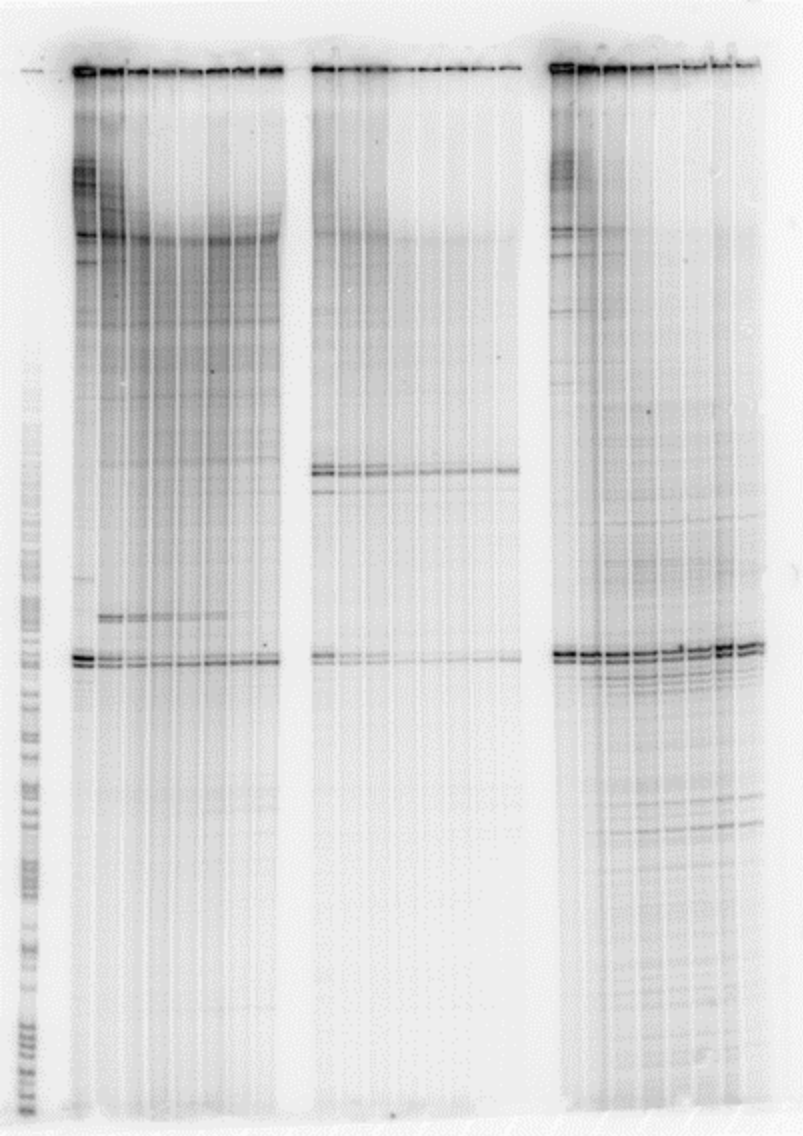

Supplement: Figure 4—source data 5. [file elife-86699-fig4-data5.pdf]

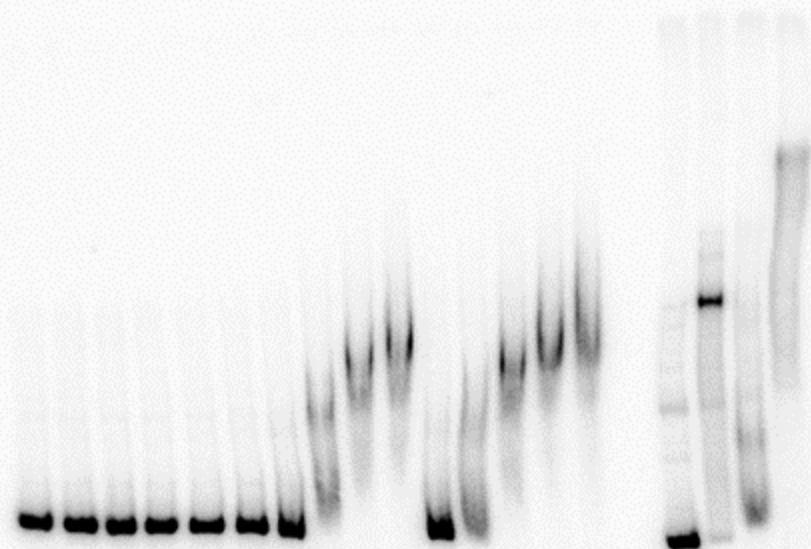

Supplement: Figure 4—figure supplement 1—source data 1. [file elife-86699-fig4-figsupp1-data1.pdf]

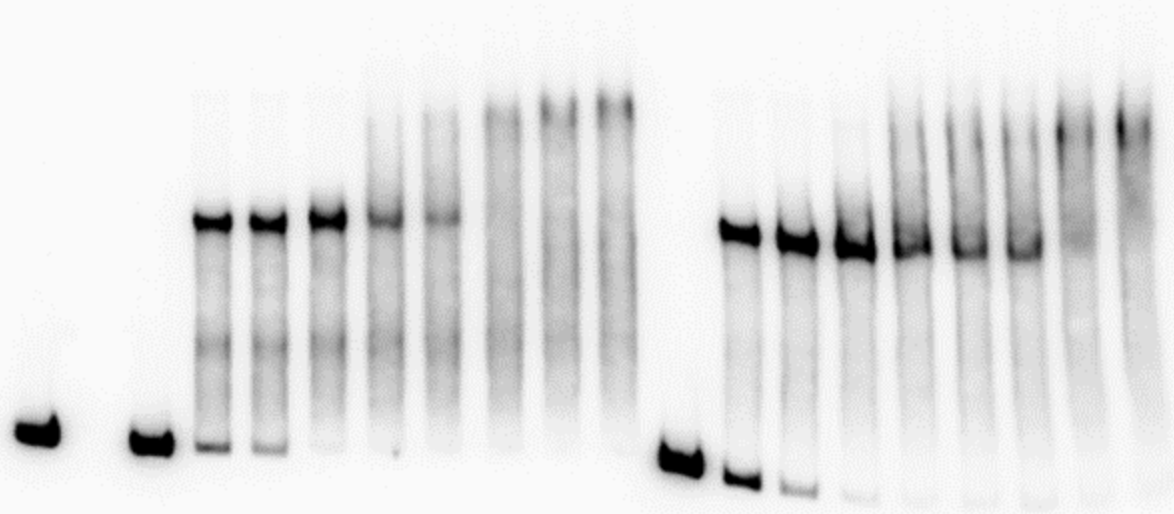

Supplement: Figure 5—source data 1. [file elife-86699-fig5-data1.pdf]

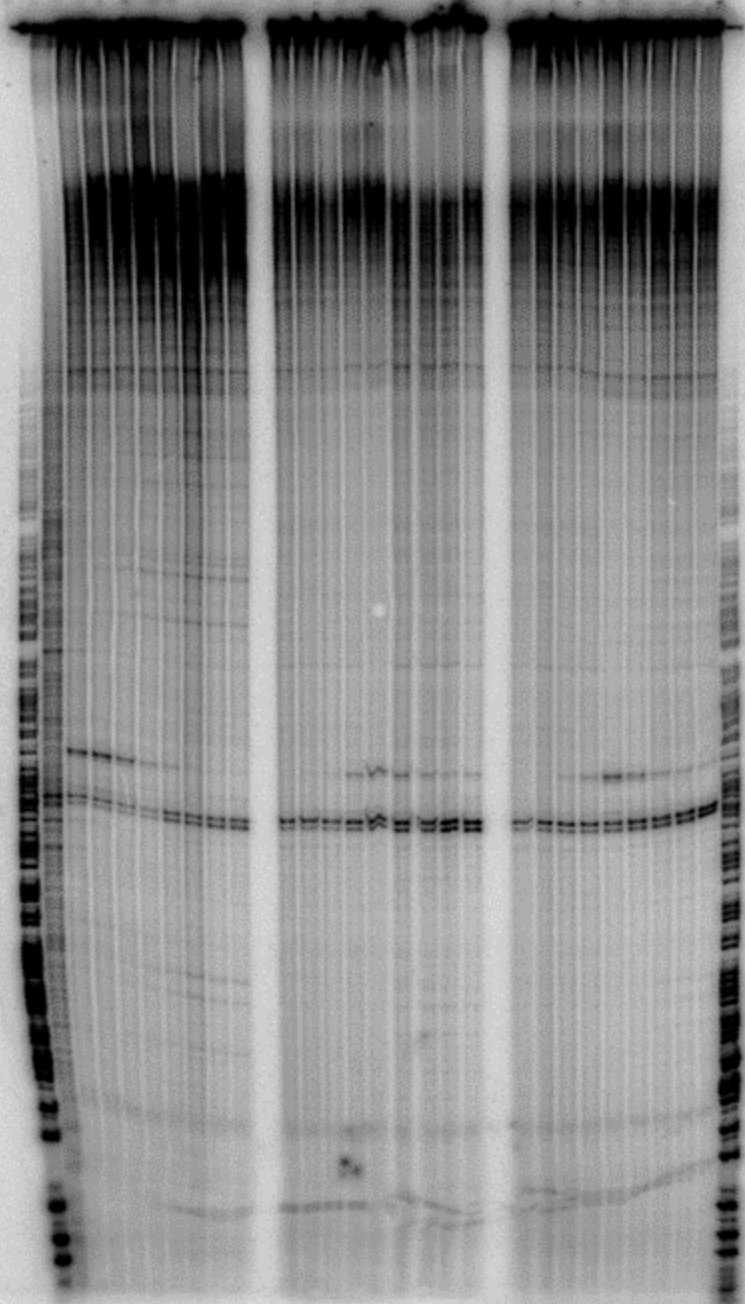

Supplement: Figure 5—source data 2. [file elife-86699-fig5-data2.pdf]

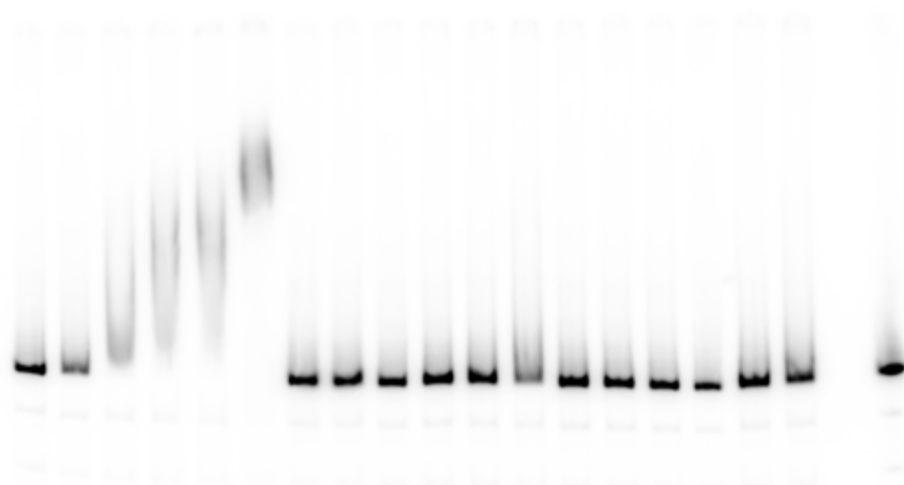

Supplement: Figure 5—figure supplement 3—source data 1. [file elife-86699-fig5-figsupp3-data1.pdf]

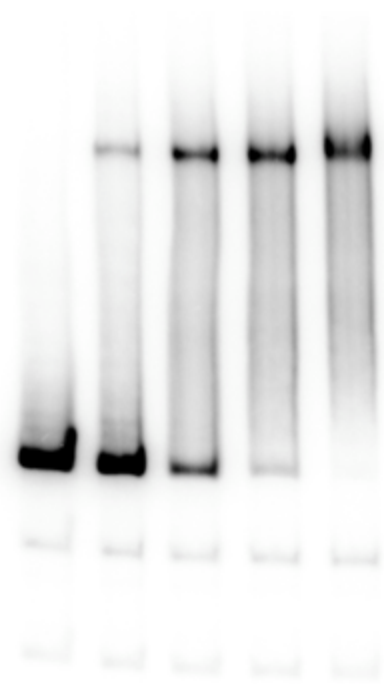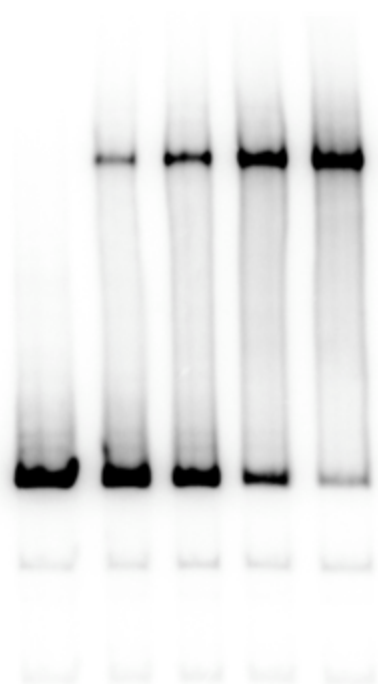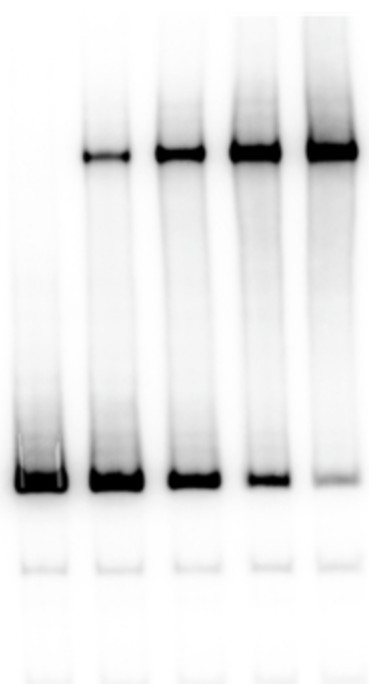

Supplement: Figure 5—figure supplement 3—source data 2. [file elife-86699-fig5-figsupp3-data2.pdf]

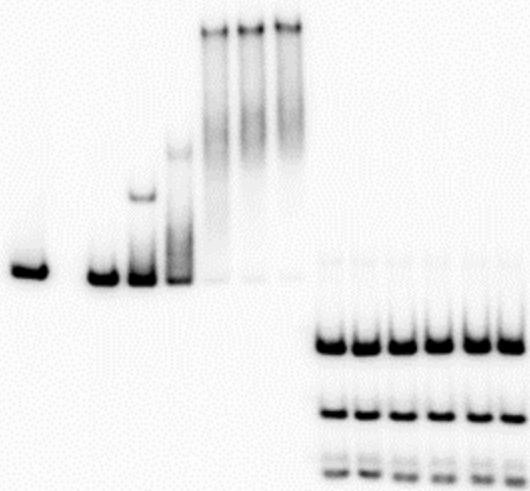

Supplement: Figure 5—figure supplement 4—source data 1. [file elife-86699-fig5-figsupp4-data1.pdf]

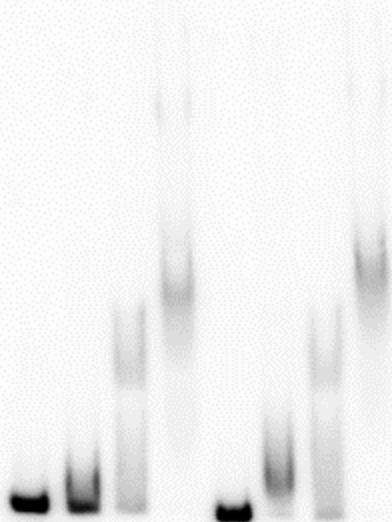

Supplement: Figure 5—figure supplement 4—source data 2. [file elife-86699-fig5-figsupp4-data2.pdf]

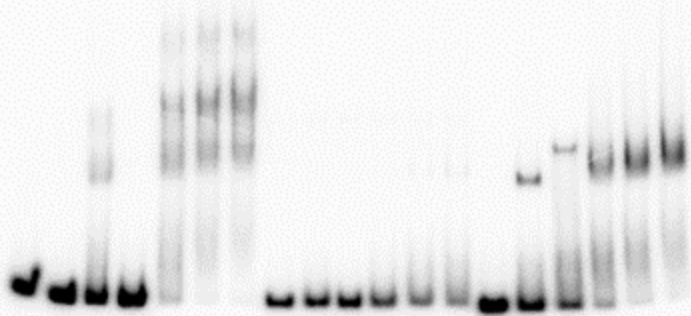

Supplement: Figure 5—figure supplement 4—source data 3. [file elife-86699-fig5-figsupp4-data3.pdf]

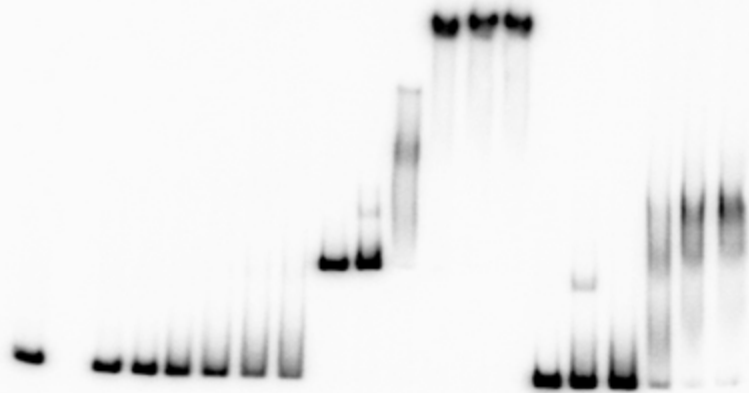

Supplement: Figure 5—figure supplement 4—source data 4. [file elife-86699-fig5-figsupp4-data4.pdf]
