# Supplementary material for: A simple mechanism for integration of quorum sensing and cAMP signalling in Vibrio cholerae
Source: eLife. 2023 Jul 6;12:RP86699. doi: 10.7554/eLife.86699 (PMC10328515; doi:10.7554/eLife.86699)

Figure 2-figure supplement 1

Figure 2b

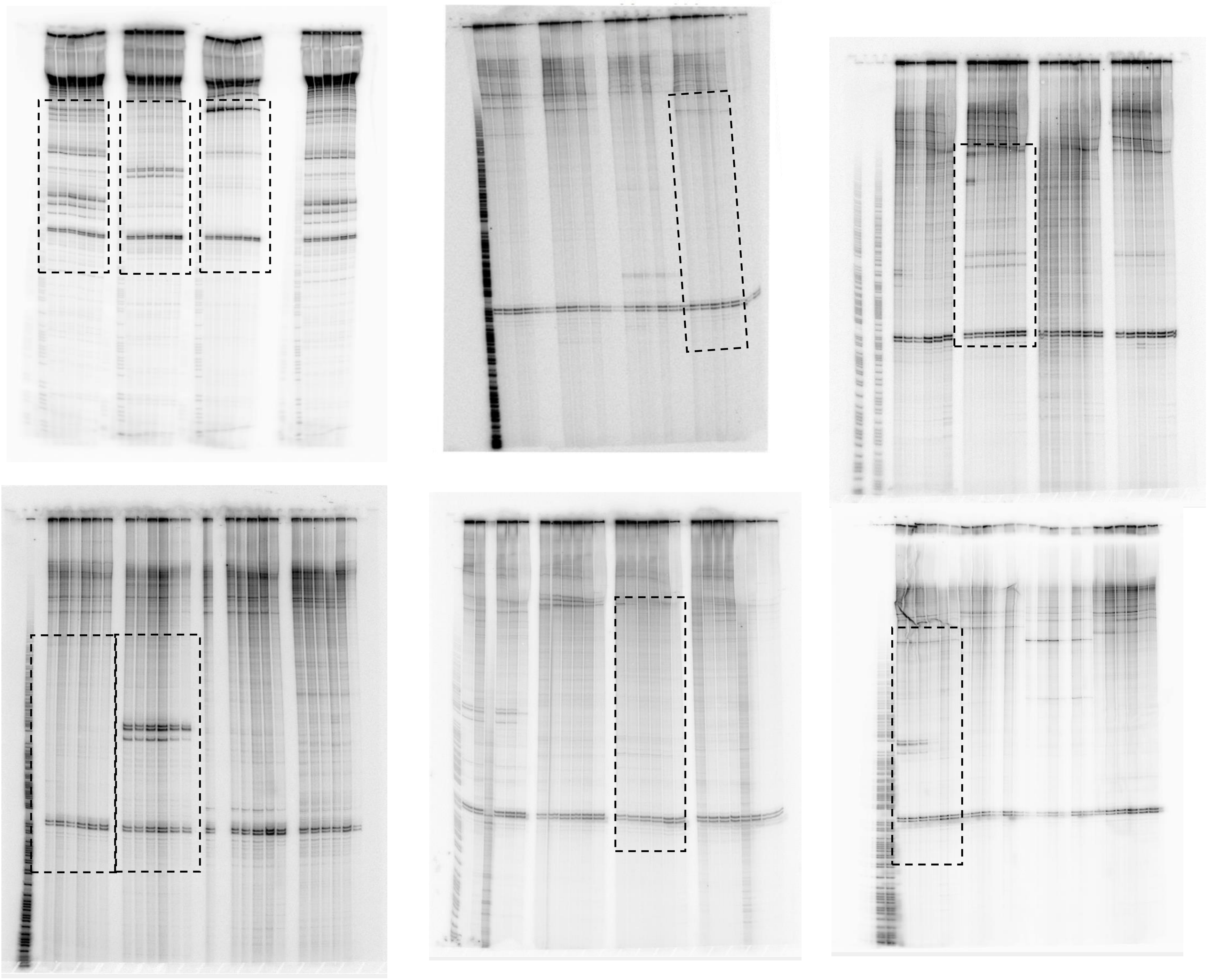

Figure 3c

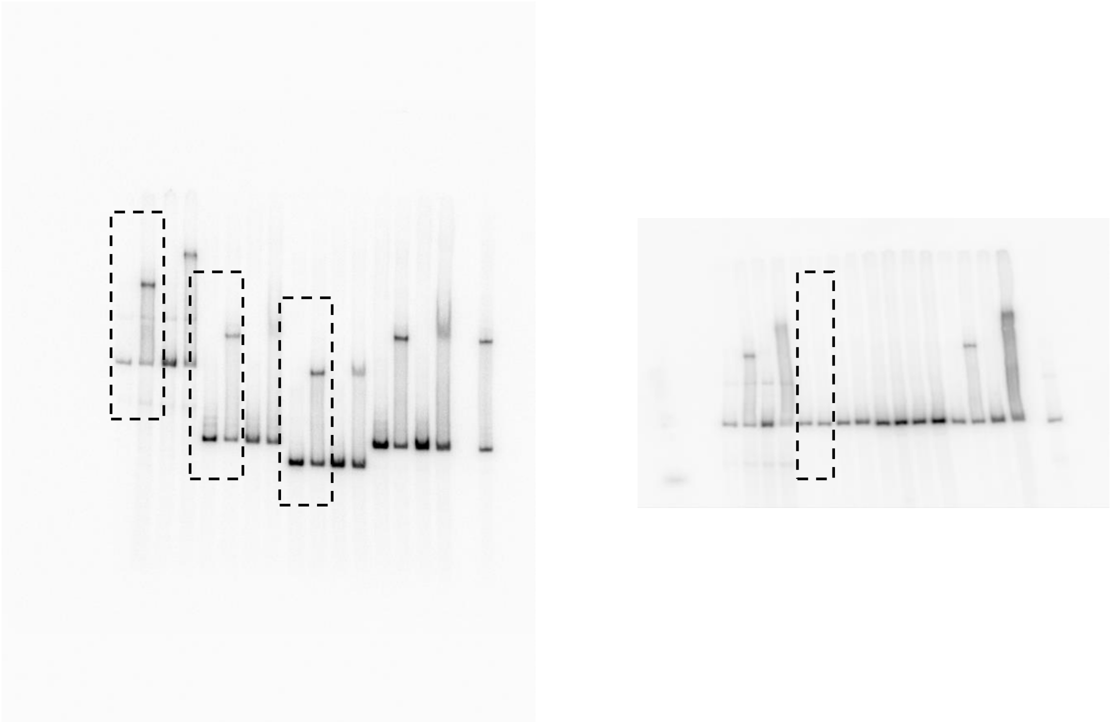

Figure 3d

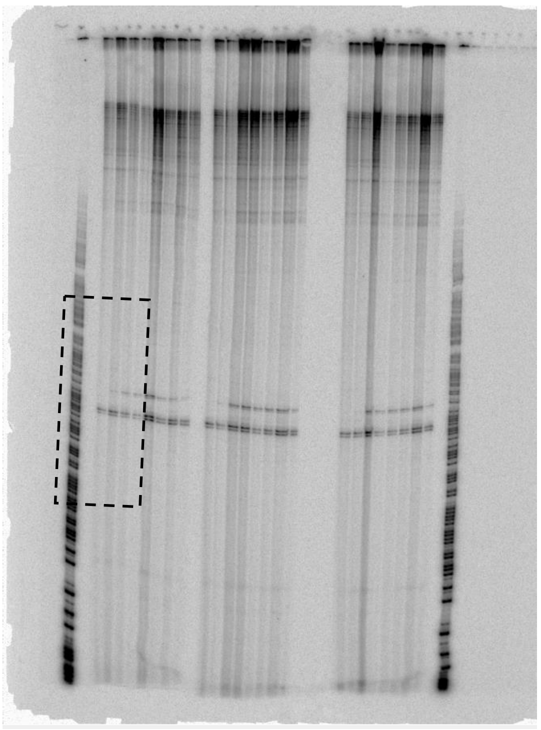

Figure 4a

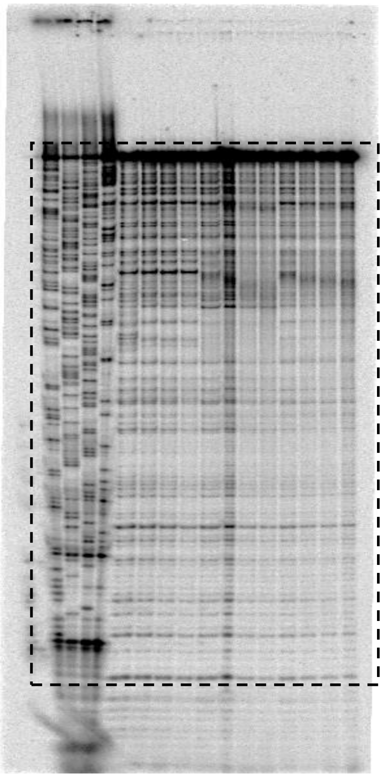

Figure 4b

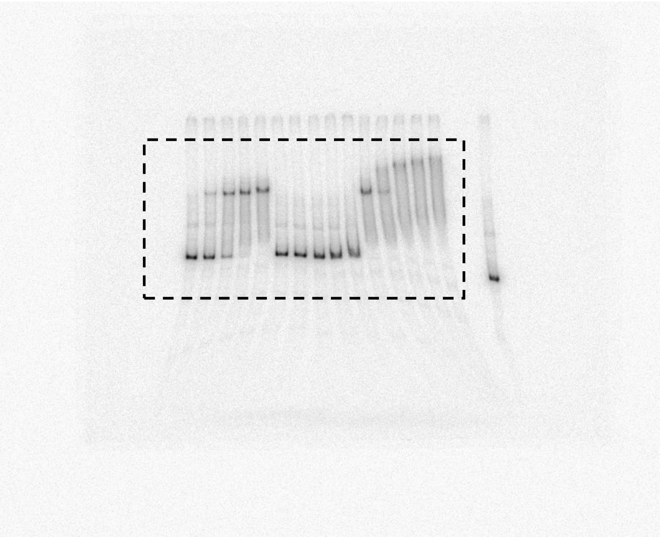

Figure 4d

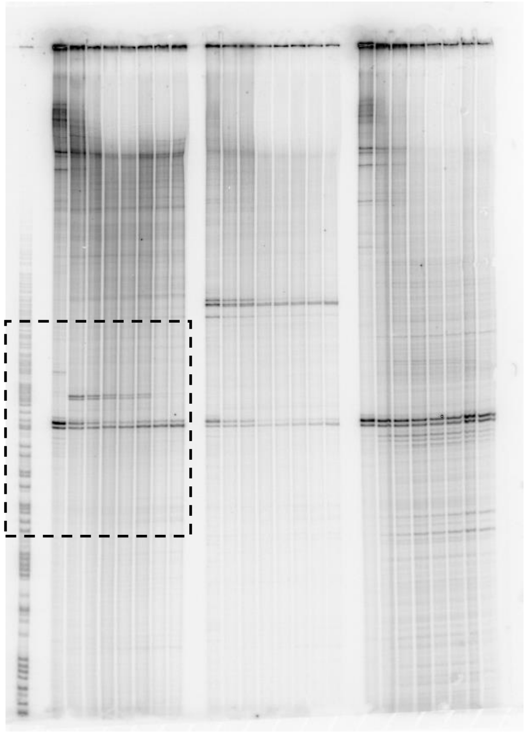

Figure 4c

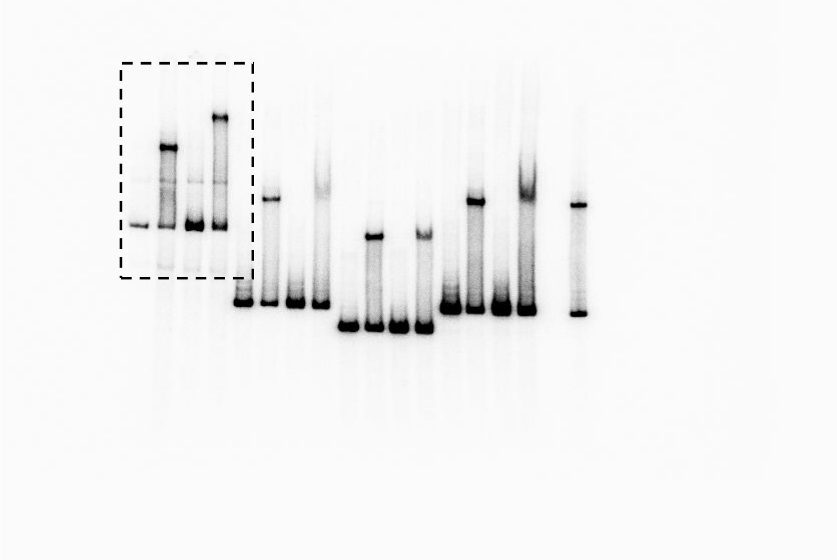

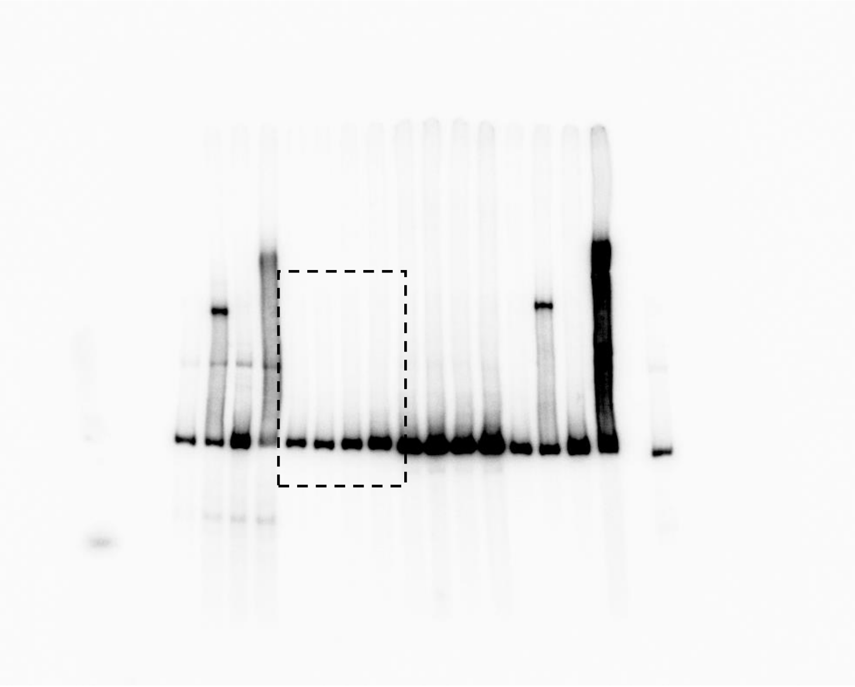

Figure 5d

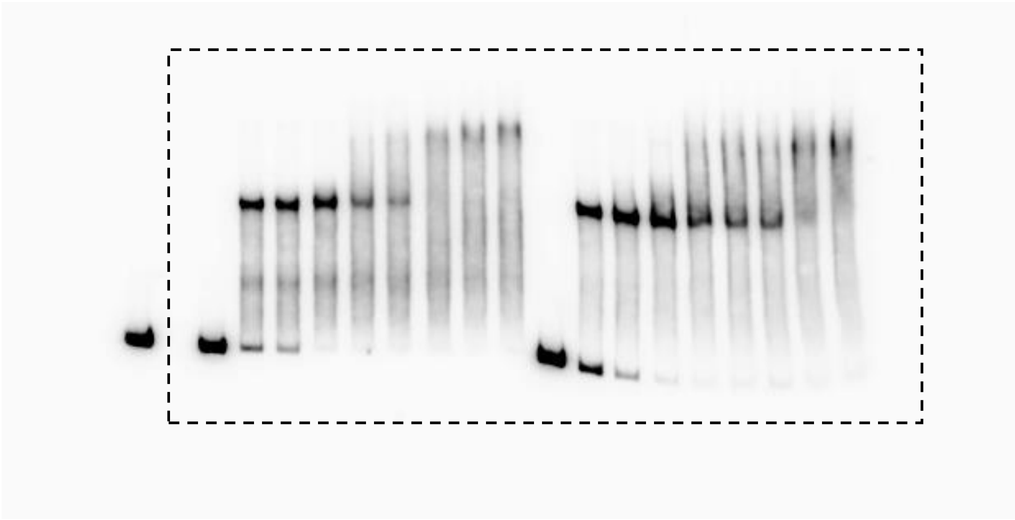

Figure 5e

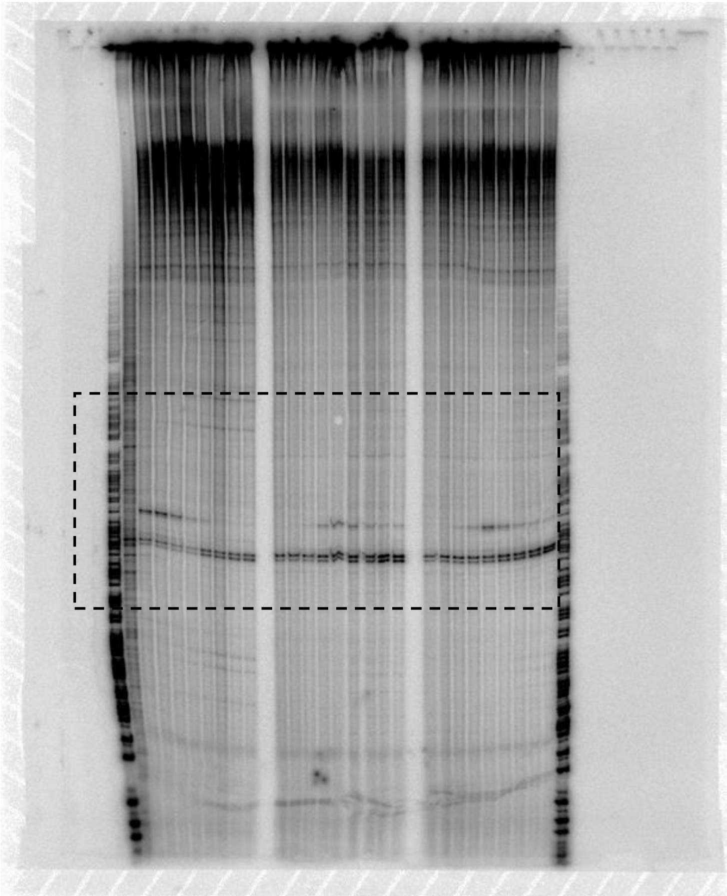

Figure 4 figure supplement 1

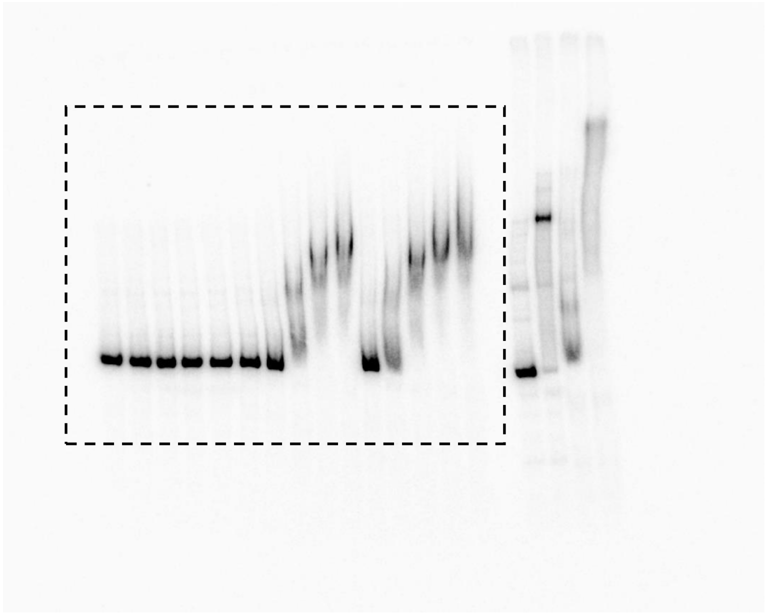

Figure 5-figure supplement 3

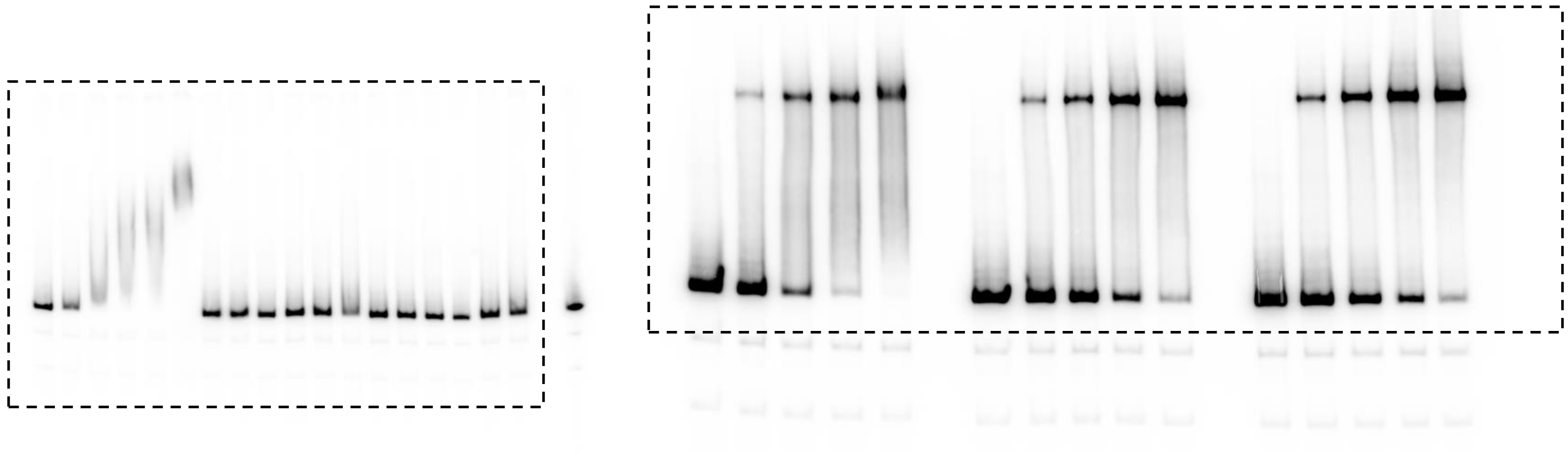

Figure 5-figure supplement 4

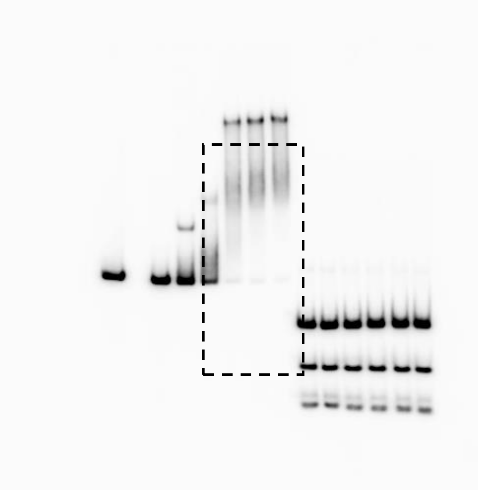

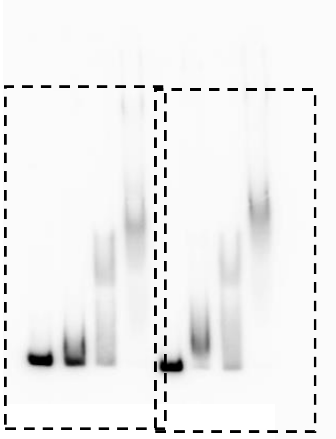

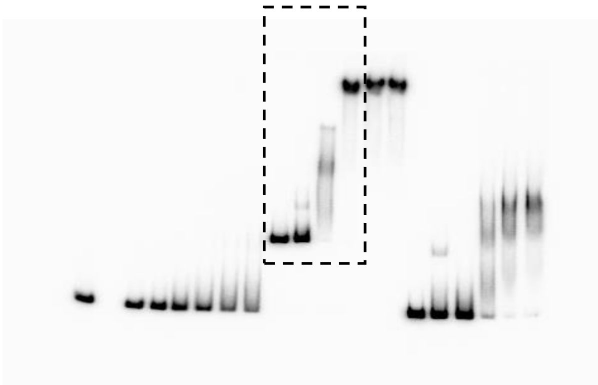

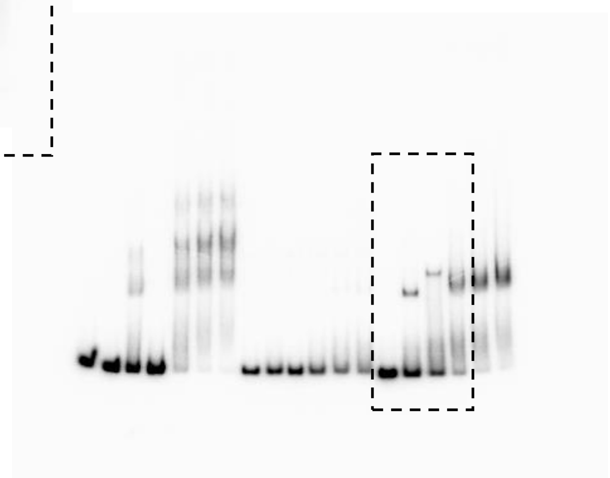

Supplement: Figure 2—source data 7. [file elife-86699-fig2-data7.pdf]
